# Variations of Tongue Coating Microbiota in Patients with Gastric Cancer

**DOI:** 10.1155/2015/173729

**Published:** 2015-09-17

**Authors:** Jie Hu, Shuwen Han, Yan Chen, Zhaoning Ji

**Affiliations:** ^1^Department of Oncology, Wannan Medical College, Wuhu, Anhui 241000, China; ^2^The Cancer Center, Yijishan Hospital of Wannan Medical College, Wuhu, Anhui 241000, China

## Abstract

The physical status of humans can be estimated by observing the appearance of the tongue coating, known as tongue diagnosis. The goals of this study were to reveal the relationship between tongue coating appearance and the oral microbiota in patients with gastric cancer and to open a novel research direction supporting tongue diagnosis. We used a tongue manifestation acquisition instrument to analyse the thickness of the tongue coating of patients with gastric cancer and that of healthy controls, and high-throughput sequencing was used to describe the microbial community of the tongue coating by sequencing the V2–V4 region of the 16S rDNA. The tongue coatings of 74 patients with gastric cancer were significantly thicker than those of 72 healthy controls (343.11 ± 198.22 versus 98.42 ± 48.25, *P* < 0.001); 51.35% of the patients were assessed as having thick tongue coatings, whereas all healthy controls were assessed as having thin tongue coatings. Thick tongue coatings presented lower microbial community diversity than thin tongue coatings. The tongue coating bacterial community is associated with the appearance of the tongue coating. The tongue coating may be a potential source for diagnosing gastric cancer, but its sensitivity needs to be further improved.

## 1. Introduction

Traditional Chinese Medicine (TCM), practiced by generations of Chinese doctors for over 3000 years [1, 2], has been abundantly used in the clinic. As an essential part of TCM, tongue diagnosis estimates human physical status by observing the appearance of the tongue coating. The normal tongue is covered by a thin, white coating that is composed of bacteria, desquamated epithelial cells, leukocytes, blood metabolites, and various nutrients. When the human body is in a morbid state, with less food and chewing, the tongue coating is thicker. Tongue diagnosis has many advantages as it is noninvasive and convenient. However, it is not widely approved due to its subjectivity and nonreproducibility because it is primarily estimated by the naked eye by experienced TCM doctors. To eliminate the influence of artificial factors, a computer-aided tongue diagnosis system has been developed to establish a uniform standard for tongue diagnosis [3, 4].

A variety of microbiota exist throughout the human body and play critical roles in the formation of various human diseases. Many studies have associated microbes in the human body with the initiation and progression of particular diseases, such as obesity [5], coronary heart disease [6, 7], and colorectal cancer [8–10]. Recently, a growing number of studies have investigated the relationship between the oral microbial community and diseases. Some studies have demonstrated that patients with pancreatic cancer [11] and patients with inflammatory bowel disease [12] have unique oral microbial community structures, and the oral microbial community may be a potential biomarker source.

Gastric cancer, as one of the most common malignancies causing death, has been considered to be the result of environmental and genetic factors. However, the specific mechanism remains obscure. It is widely accepted that* Helicobacter pylori*, which is commonly found in oral cavity, is a strong inducer of gastric cancer and precancerous lesions [13–15]. Epidemiological studies have also implicated human oral bacteria in the aetiology of gastrointestinal cancers. Much evidence has implicated oral microbiota. Previously, technological limitations prevented the description of complicated oral microbes. The development of next-generation sequencing technology makes it possible to comprehensively describe the microbiota in the oral cavity.

In the present study, a tongue manifestation acquisition instrument was used to compare the thickness of the tongue coating of patients with gastric cancer and that of healthy controls. In addition, next-generation sequencing was used to describe the tongue coating microbiota of patients with different thicknesses of tongue coatings and healthy controls.

## 2. Materials and Methods

### 2.1. Individual Screening

Seventy-four patients with histologically confirmed gastric cancer (GC) in Yijishan Hospital (Wannan Medical College First Affiliated Hospital) from November 2013 to April 2014 and 72 volunteers who had no stomach discomfort over the past three years were recruited as cases and healthy controls (HCs), respectively. The HCs had no malignant tumours, oral diseases, or gastric diseases, and the absence of oral disease among patients with GC was confirmed. To remove chemotherapeutics and surgery as confounding variables for tongue coating microbiota, all patients selected had been diagnosed for 1 day to 26 days and had not undergone any chemotherapy or surgery. None of the subjects had used any antibiotics within the past two months. The use of these subjects was approved by the Yijishan Hospital. All patients and healthy controls in the study provided their informed consent.

### 2.2. Tongue Image Analysis

All tongue images were photographed in the morning prior to patient food consumption to avoid the interference of food debris, and the thickness of the tongue coatings was analysed by the DS01-B tongue manifestation acquisition instrument (Daosh Co., Shanghai, China). The DS01-B tongue manifestation acquisition instrument consists of a photographic system and a software analysis system that can quantify the thickness of tongue coatings automatically. This instrument has been applied and proven effective in a previous research study [16].

### 2.3. Sample Collection

All subjects were required to rinse their mouths with saline buffer before sample collection. The tongue coating samples were collected by scraping the tongue surface with 3 sterile swabs 3 times. Then, the sterile swabs were soaked in tubes with 1 mL of phosphate-buffered saline to wash off the tongue coating adsorbed on the sterile swabs. The tubes were centrifuged for 5 min at 5000 rpm, and the precipitates were collected. Samples were frozen in liquid nitrogen and immediately stored at −80°C.

### 2.4. Gene Amplification

DNA was extracted using Tiangen extraction kits (Tiangen, Peking, China) according to the manufacturer's protocols. The integrity of the genomic DNA was assessed by electrophoresis (1% agarose gel). We amplified the V2–V4 region (338F-806R) of the 16S rRNA using universal primers [17]. All samples were amplified on an ABI GeneAmp 9700 (ABI, USA) using the following parameters: 94°C for 10 min, then 30 cycles of 94°C for 30 sec, 55°C for 30 sec, and 72°C for 60 sec, with a final incubation at 72°C for 10 min. PCR reactions were performed in triplicate in a 20 *μ*L mixture containing 4 *μ*L of 5× FastPfu Buffer, 2 *μ*L of 2.5 mM dNTPs, 0.8 *μ*L of each primer (5 *μ*M), 0.4 *μ*L of FastPfu Polymerase, and 10 ng of template DNA.

### 2.5. Illumina Sequencing

Amplicons were extracted from 2% agarose gels and purified using the AxyPrep DNA Gel Extraction Kit (Axygen Biosciences, Union City, CA, USA) according to the manufacturer's instructions and quantified using QuantiFluor-ST (Promega, USA). Purified amplicons were pooled in equimolar amounts and paired-end sequenced (2 × 300) on an Illumina MiSeq platform according to the standard protocols.

### 2.6. Sequence Analysis

To obtain accurate data, we eliminated reads with more than 2 mismatched bases in the forward primer, cut off low-quality bases from the 3′ end, and discarded reads with ambiguous bases, short reads, or reads with an average accuracy less than 0.2. Filtered sequences were binned into OTUs with the QIIME pipeline. The OTU is an artificial classification unit utilized in phylogenetics and population genetics studies. OTU clustering is conducted to estimate the number of species or genera in each sample by applying a similarity threshold (97% in our study). To ensure accuracy, a UPARSE pipeline was used to cluster sequences into OTUs [18], and UCHIME was used to eliminate chimaeras among sequences [19]. To perform the taxonomic analysis, each OTU was aligned to the SILVA database [20], and the closest species or genera with confidence levels higher than 70% were identified. We analysed population structure and richness based on each of the following five categories: phylum, class, order, family, and genus.

### 2.7. Statistical Analysis

An independent *t*-test was used to calculate relative abundance. Frequency table data were analysed using *χ* ^2^ analysis or Fisher's exact test. Differences between the groups were evaluated using the Student-Newman-Keuls (SNK) test. All statistical tests were two-sided, and a *P* value less than 0.05 was considered statistically significant.

## 3. Results

### 3.1. General Characteristics and Tongue Coating Thickness

**Figure 1:**
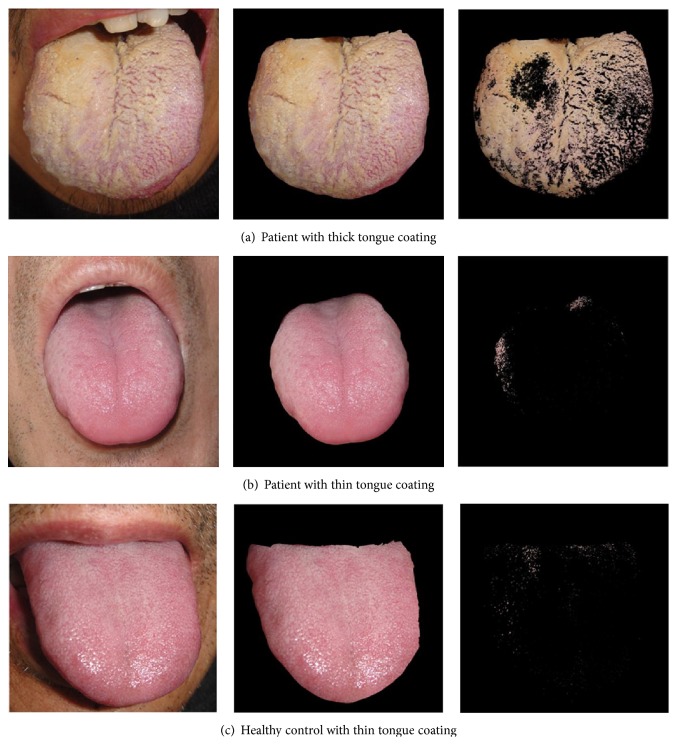
Images of tongue coatings analysed by a tongue manifestation acquisition instrument. (a) A patient with a typical thick tongue coating, (b) a patient with a thin tongue coating, and (c) a healthy control with a thin tongue coating are shown.

We used the DS01-B tongue manifestation acquisition instrument to analyse the thickness of tongue coatings and found that the thickness differed between the patients and the healthy controls. To control for factors that may influence the microbiota of tongue coatings, we included BMI [21], diabetes [22], and hypertension [6], which may influence the human body microbial community, and smoking [23] and drinking [24], which may be associated with the risk of gastric cancer, in the statistical analysis. As shown in Table 1, HCs did not significantly differ from GC patients with respect to age, gender, BMI, smoking, drinking, diabetes, or hypertension (*P* > 0.05); however, the thickness of the tongue coating was significantly different between the two groups (*P* < 0.001). According to the manual of the DS01-B tongue manifestation acquisition instrument, tongue coatings were divided into thick tongue coatings and thin tongue coatings at the threshold of 300. Tongue coatings valued above 300 were regarded as thick tongue coatings, and coatings with values below 300 were considered thin tongue coatings.  Figure 1 is an image analysed by the tongue manifestation acquisition instrument. Using the tongue manifestation acquisition instrument, the tongue coatings of all HCs (value range: 28.8–218.7) and those of 36 of the 74 patients (48.6–288.3) were determined to be thin, whereas the coatings of 38 of the 74 patients (310.1–732.2) were considered thick (Table 2). Though all healthy controls had thin tongue coatings, 48.65% of patients also had thin tongue coatings, indicating that nearly half of the GC patients could not be detected by the tongue manifestation acquisition instrument. Therefore, improving the sensitivity and reliability of this tool is a vital challenge for the future.

### 3.2. Diversity of the Microbial Community in Different Thicknesses of Tongue Coatings

To obtain better samples for the next-generation sequencing, 40 of 74 patients and 56 of 72 healthy controls were eliminated because of nonstandard operation, resulting in 34 patients and 17 healthy controls as the final research subjects. The tongue coatings of all HCs (value range: 37.2–190.4) and 16 of 34 patients (54.2–273.3) were determined to be thin, whereas the coatings of 18 of 34 patients (310.1–699.2) were considered thick. Therefore, there were the three following groups: HCs with thin tongue coatings (*n* = 16), patients with thin tongue coatings (*n* = 16), and patients with thick tongue coatings (*n* = 18).

To estimate the diversity of the microbial community in the tongue coatings of the three groups, abundance-based coverage estimator (ACE), Chao, and Shannon indices were used to describe the alpha diversity. We compared the alpha diversities of these three groups and found that the number of OTUs in the thick tongue coating group was significantly lower than those of the thin tongue coating group and the control group. The ACE, Chao, and Shannon values showed similar results (Table 3). These findings indicate that patients with thick tongue coatings have lower microbial community diversity than patients and healthy people with thin tongue coatings.

### 3.3. Relative Abundances of Microbes in the Three Groups

**Figure 2:**
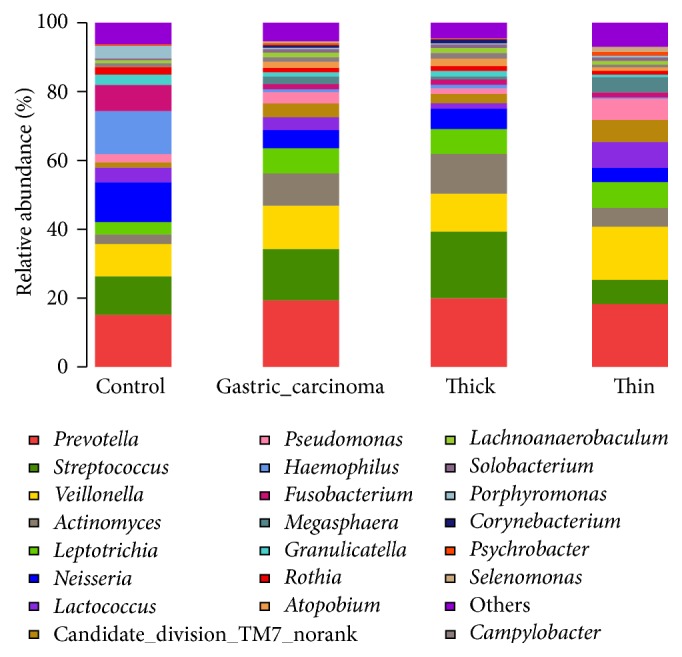
Relative abundance at the genera level. The five most abundant genera found in patients with thin tongue coatings were* Prevotella*,* Veillonella*,* Leptotrichia*,* Lactococcus*, and* Streptococcus*, and the five most abundant genera in patients with thick tongue coatings were* Prevotella*,* Streptococcus*,* Actinomyces*,* Veillonella*, and* Leptotrichia*.

An image of the relative abundances of bacteria at the genus level in each of the three groups was produced (Figure 2). The five genera with the greatest relative abundances in patients with thin tongue coatings were* Prevotella*,* Veillonella*,* Leptotrichia*,* Lactococcus*, and* Streptococcus*, and those of patients with thick tongue coatings were* Prevotella*,* Streptococcus*,* Actinomyces*,* Veillonella*, and* Leptotrichia*. Patients with thick tongue coatings had higher relative abundances of* Actinomyces* and* Streptococcus* than the other two groups.

### 3.4. Species on the Tongue Coating

**Figure 3:**
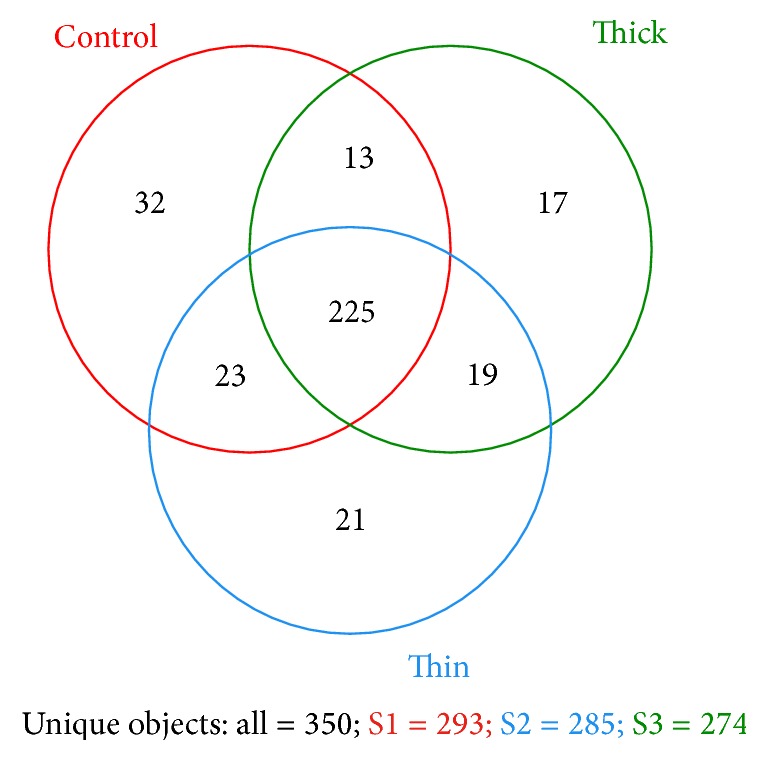
A Venn diagram showing the species-level diversity of the three groups. Two hundred and twenty-five species were shared by three groups, 32 species were not detected in patients, 47 species were not detected in healthy controls, and 17 species were detected only in thick tongue coatings.

We also investigated the microbial diversity at the species level. The majority of species were shared among the three groups. A Venn diagram of the diversity at the species level showed that 225 species were shared among the three groups, 32 species were not detected in patients, 47 species were not detected in healthy controls, and 17 species were detected only in thick tongue coatings (Figure 3). However, the diagram suggests that each individual had his or her own characteristic species profile.

### 3.5. The Tongue Coating Microbial Community of Patients and Controls

**Figure 4:**
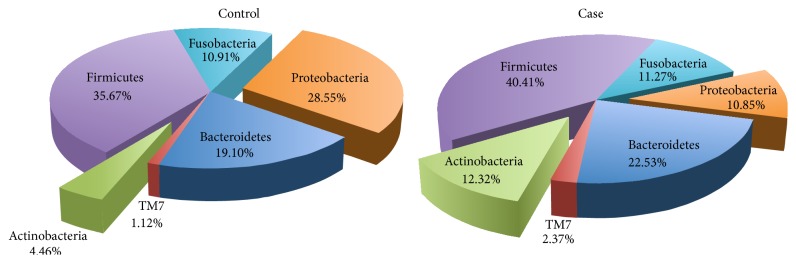
Relative abundances of dominant phyla in cases and controls. The dominant phyla were Firmicutes, Proteobacteria, Bacteroidetes, Actinobacteria, Fusobacteria, and TM7. The abundance of Proteobacteria was 28.55% in controls compared with 10.85% in cases, whereas the abundance of Actinobacteria was 4.46% in controls compared with 12.32% in cases.

We compared the tongue coating microbial community of patients and controls. Both patients and controls contained six dominant phyla, Firmicutes, Proteobacteria, Bacteroidetes, Actinobacteria, Fusobacteria, and TM7, which accounted for 99% of the tongue coating microbes. The relative abundances of Firmicutes, Bacteroidetes, Fusobacteria, and TM7 were similar between patients and controls. However, there were significant differences in the abundances of Proteobacteria (10.85% versus 28.55% relative abundance for patients and controls, resp., *P* < 0.001) and Actinobacteria (12.32% versus 4.46%, resp., *P* < 0.001), as shown in Figure 4. Proteobacteria accounted for 28.55% of the tongue coating microbes from GC patients (ranging from 11.70% to 42.99%) and 10.85% of the tongue coating microbes from HCs (ranging from 0.11% to 34.77%). Relative abundance levels of less than 15% were determined for Proteobacteria in only 1 of 16 GC patients but in 25 of 34 HCs.

At the genus level, 162 genera were identified; the five genera with the highest relative abundances in patients were* Prevotella*,* Streptococcus*,* Veillonella*,* Actinomyces*, and* Leptotrichia*, whereas those in controls were* Prevotella*,* Neisseria*,* Streptococcus*,* Haemophilus*, and* Fusobacterium*. Compared with the controls, patients had lower relative abundance levels of* Fusobacterium* (1.78% versus 6.43%, *P* = 0.004),* Neisseria* (4.67% versus 10.97%, *P* = 0.008),* Haemophilus* (1.36% versus 7.46%, *P* = 0.007), and* Porphyromonas* (0.34% versus 3.43%, *P* = 0.002) (Table 4).

## 4. Discussion

This study is among the first to systematically profile the microbiota in tongue coating samples of patients with GC. In the present study, we found that the tongue coatings of patients with GC were significantly thicker than those of HCs, the tongue coating microbiota community was correlated with the appearance of the tongue coating, and patients with thick tongue coatings had decreased microbial community diversity compared with those of patients and healthy people with thin tongue coatings. These observations may promote tongue coating as a potential diagnostic resource for gastric cancer, which should be sufficiently noninvasive and inexpensive to allow widespread applicability. Compared with other invasive examinations, tongue coating examinations are easier to conduct and may be better accepted by patients.

Compared with previous studies [25–27], we obtained consistent results for the following six dominant phyla from our samples: Firmicutes, Proteobacteria, Bacteroidetes, Actinobacteria, Fusobacteria, and TM7. Our data showed significant differences in the tongue coating microbiota composition between GC patients and HCs. Analysis at the phyla level revealed that the relative abundance of Proteobacteria was significantly lower in GC patients than in HCs (*P* < 0.001), which was mainly attributed to lower abundances of* Neisseria* and* Haemophilus*. This finding is consistent with results from a previous study [12].* Fusobacterium* and* Porphyromonas*, which contribute to periodontal disease [28, 29], were also less abundant in GC patients than in HCs. Their prevalence was reported to have an impact on the risk of colorectal cancer [30]. Our study describing the tongue coating microbial community of patients with GC may lead to the development of tongue coating analysis as a microbe-related tool for the early diagnosis of gastric cancer.

The relationship between oral microbiota and gastric cancer should be deeply investigated. Some former studies have indicated that some factors, such as poor oral hygiene [31], tooth loss [32, 33], and the metabolism of oral microbes [34], may influence the risk of gastric cancer [35]. In future studies, we will further investigate the interplay of oral microbiota and gastric microbiota, which may become a biological approach for gastric cancer prevention.

In our study, a computer-aided tongue diagnosis system was used to measure the thickness of tongue coatings. Although the system has many advantages, its reliability is questionable. Nearly half of GC patients could not be detected by the system; therefore, the system can serve as only an assisted screening method in clinical work. It cannot serve as an independent and standard diagnostic method unless its sensitivity is greatly improved.

Our study also has some limitations. First, the limited sample size may have caused bias due to human sample variation, sample preparation techniques, and other existing medical conditions. Second, approximately half of the patients with GC in our study possessed thin tongue coatings similar to those of healthy people; therefore, improving the accuracy of tongue coating evaluation to serve as an early diagnosis tool of GC is a serious challenge. Further investigations should use higher sample sizes and aim at revealing how microbes participate in diseases. Regardless, as there is a lack of breakthroughs in cancer screening, analysis of tongue coating microbiota could be an innovative source for gastric cancer screening and diagnosis.

## Figures and Tables

**Table 1:** General characteristics and tongue coating thickness of all subjects.

	Normal controls (*n* = 72)	Gastric cancer patients (*n* = 74)	*P* ^§^
Male, *n*	35	37	0.834
Age (mean ± SD), years	54.55 ± 9.63	57.46 ± 8.43	0.464
BMI (mean ± SD), kg/m^2^	22.16 ± 2.14	23.13 ± 3.14	0.530
Habit of smoking, *n*	12	15	0.848
Habit of drinking, *n*	15	16	0.902
Diabetes, *n*	1	2	0.959
Hypertension, *n*	5	5	0.850
Thickness of the tongue coating	98.42 ± 48.25	343.11 ± 198.22	<0.001

^§^ *P* values were based on *t*-tests (two-sided) and *χ* ^2^ analysis or Fisher's exact tests.

**Table 2:** The number of controls and patients with thin and thick tongue coatings.

	Thin tongue coating (%)	Thick tongue coating (%)	Total
Normal controls	72 (100)	0 (0)	72
Patients with gastric cancer	36 (48.65)	38 (51.35)	74
Total	108	38	146

**Table 3:** Tongue coating thickness and the alpha diversity of three groups.

	OTU (mean ± SD), *n*	ACE (mean ± SD)	Chao (mean ± SD)	Shannon (mean ± SD)
Normal controls	153.31 ± 30.36	205.39 ± 37.88	198.05 ± 34.97	3.06 ± 0.26
Thin tongue coating group	151.69 ± 37.68^*∗*^	197.54 ± 11.77^*∗*^	187.68 ± 11.13^*∗*^	3.16 ± 0.10^*∗*^
Thick tongue coating group	116.06 ± 36.83^#^	160.23 ± 14.73^#^	151.21 ± 12.48^#^	2.85 ± 0.10^#^
*P* ^§^	0.001	0.019	0.003	0.05

^§^ *P* values were based on ANOVA.

^*∗*^ *P* > 0.05 compared with the control group, ^#^ *P* < 0.05 compared with the control group and the thin tongue coating group (based on SNK).

**Table 4:** Relative abundances of selected tongue coating microbial taxa in 34 gastric cancer subjects and 16 control subjects.

Taxa (phylum, class, order, family, and genus)	Abundance %		
	Case	Control	*P* ^§^
Proteobacteria (phylum)	10.85	28.55	<0.001
Proteobacteria, Betaproteobacteria, Neisseriales, Neisseriaceae, and *Neisseria *(genus)	4.67	10.97	0.008
Proteobacteria, Gammaproteobacteria, Pasteurellales, Pasteurellaceae, and *Haemophilus *(genus)	1.36	7.46	0.007
Actinobacteria (phylum)	12.32	4.46	<0.001
Fusobacteria, Fusobacteria, Fusobacteriales, Fusobacteriaceae, and* Fusobacterium *(genus)	1.78	6.43	0.004
Bacteroidetes, Bacteroidia, Bacteroidales, Porphyromonadaceae, and* Porphyromonas *(genus)	0.34	3.43	0.002

^§^ *P* values were based on *t*-tests (two-sided).
